# Walton’s Operative Opthalmic Surgery

**Published:** 1853-12

**Authors:** 


*Walton's Operative Ophthalmic Surgery.* Littel. Published by Lindsay & Blackiston, Phila.

We have long been in want of a work on the operative surgery of the eye, which should embrace all the latest additions to that branch, and this one is eminently fitted to fill the void, for it is full and concise, and strictly up to the time. Chloroform, which has been so useful in operations on other parts of the body, has strangely been neglected in those about the eye, and we are glad to see that Mr. Walton advocates its use, where steadiness is so desirable. He uses it in all cases, except in cataract by extraction, or wounds of the cornea to a similar extent. In iritis, instead of the old plan of giving calomel to an alarming extent, Mr. Walton is moderate in his use of mercury, and says he has had fully as good an effect from the use of two or three grains of the hydragyrum cum creta, three times a day, without any of the after bad consequences. We never yet believed that transplanting either the cilia or the cornea was attended with good results, and although he speaks of both operations, their performance was not attended with success. Altogether, we like the book, and it is full of useful hints in relation to the treatment of accidents to which the eye is liable, and for which, heretofore, it was necessary to look over so many works. M.

